# Clinicopathologic and ultrasonographic features of combined hepatocellular-cholangiocarcinoma and its correlation with microvascular invasion: a predictive role of contrast-enhanced ultrasound

**DOI:** 10.3389/fonc.2024.1474675

**Published:** 2024-12-20

**Authors:** HaiYing Tian, Yuling Chen, LiNa Zhao, ChunYan Liao, Sha Li, Bei Zhang

**Affiliations:** ^1^ Clinical Medical College, Guizhou Medical University, Guiyang, Guizhou, China; ^2^ Department of Ultrasound Medicine, Guizhou Provincial People’s Hospital, Guiyang, Guizhou, China; ^3^ National Health Commission (NHC) Key Laboratory of Pulmonary Immune-Related Diseases, Guizhou Provincial People’s Hospital, Guiyang, Guizhou, China; ^4^ Department of Pathology, Guizhou Provincial People’s Hospital, Guiyang, Guizhou, China; ^5^ Department of Ultrasound Medicine, The Affiliated Hospital of Guizhou Medical University, Guiyang, Guizhou, China

**Keywords:** combined hepatocellular-cholangiocarcinoma, microvascular invasion, contrast-enhanced ultrasound, risk factors, primary liver cancer

## Abstract

**Background:**

This study aims to investigate the clinicopathological and ultrasonography characteristics of combined hepatocellular-cholangiocarcinoma (cHCC-CCA) and its correlation with microvascular invasion (MVI), as well as the predictive value of contrast-enhanced ultrasound (CEUS) imaging.

**Methods:**

A retrospective analysis was conducted on 57 patients diagnosed with cHCC-CCA between November 2017 and May 2023 at Guizhou Provincial People’s Hospital. Among them, 27 patients were MVI-positive and 30 patients were MVI-negative, all of whom underwent preoperative CEUS within 2 weeks. Clinical data, ultrasonographic findings, and CEUS features were compared between the two groups to analyze the influencing factors and predictive value of MVI in cHCC-CCA patients.

**Results:**

Compared to the MVI-negative group, the MVI-positive group showed a higher proportion of tumors with a maximum diameter greater than 5 cm, elevated alpha-fetoprotein (AFP) levels, low echo halo around the tumor, non-smooth tumor contour, peripheral irregular rim-like enhancement and early washout (≤60s) with nodular patterns on CEUS (P<0.05). Multivariate logistic regression analysis revealed that low echo halo, peripheral irregular rim-like enhancement, and early washout were independent risk factors for MVI in cHCC-CCA patients. The receiver operating characteristic (ROC) curve analysis demonstrated an area under the curve (AUC) of 0.8056 for these factors.

**Conclusions:**

Ultrasonographic and CEUS features have a certain correlation with MVI in cHCC-CCA patients. Low echo halo, peripheral irregular rim-like enhancement, and early washout are independent risk factors for MVI in patients with cHCC-CCA. These features have a predictive value in determining the presence of MVI in patients with cHCC-CCA.

## 1 Introduction

Combined hepatocellular-cholangiocarcinoma (cHCC-CCA) is a primary liver cancer (PLC) with heterogeneous phenotypes that share common characteristics of both hepatocytic and cholangiocytic differentiation (1). cHCC-CCA is rare, with reported incidences ranging from 0.4% to 14.2% of PLCs. The World Health Organization (WHO) estimates a similar incidence at 2%–5% of PLCs (2–4). The cHCC-CCA was initially described by Allen and Lisa in 1949, nevertheless, the demographic and clinical features of these tumors remain ambiguous.

Microvascular invasion (MVI) serves as an indicator of tumor invasiveness and is an adverse prognostic factor associated with early disease recurrence and lower survival rates (5–7). MVI is characterized by the infiltration of tumor cells into small blood vessels surrounding the tumor, such as the portal vein and hepatic vein systems, indicating a more aggressive biological behavior. Some scholars posit that MVI is the first step in the development of intrahepatic or systemic metastasis in liver cancer (8, 9). Consequently, preoperative prediction of MVI would facilitate treatment planning and enhance prognosis. Hence, some researchers suggest that patients with cHCC-CCA who are predicted to have MVI should undergo anatomical liver resection, expanding the scope of lesion removal to reduce early recurrence rates (10–12). Therefore, early and accurate assessment of MVI has significant implications for treatment decisions and prognosis prediction in cHCC-CCA patients. Unfortunately, the confirmation of MVI mostly depends on histopathological examination of surgical specimens. Currently, there are limited reports on the predictive role of preoperative contrast-enhanced ultrasound (CEUS) in detecting MVI in cHCC-CCA patients (13). However, CEUS can reflect the blood perfusion of tumor tissue in real time, which has important clinical value in the diagnosis of focal liver lesions. In addition, due to the relatively small sample size, the imaging features of cHCC-CCA on CEUS and its relationship with histopathological features are not well summarized. Hence, the objective of this study is to analyze the prediction of MVI in cHCC-CCA using preoperative CEUS and clinicopathological features.

## 2 Methods

### 2.1 Patients

**Figure 1:**
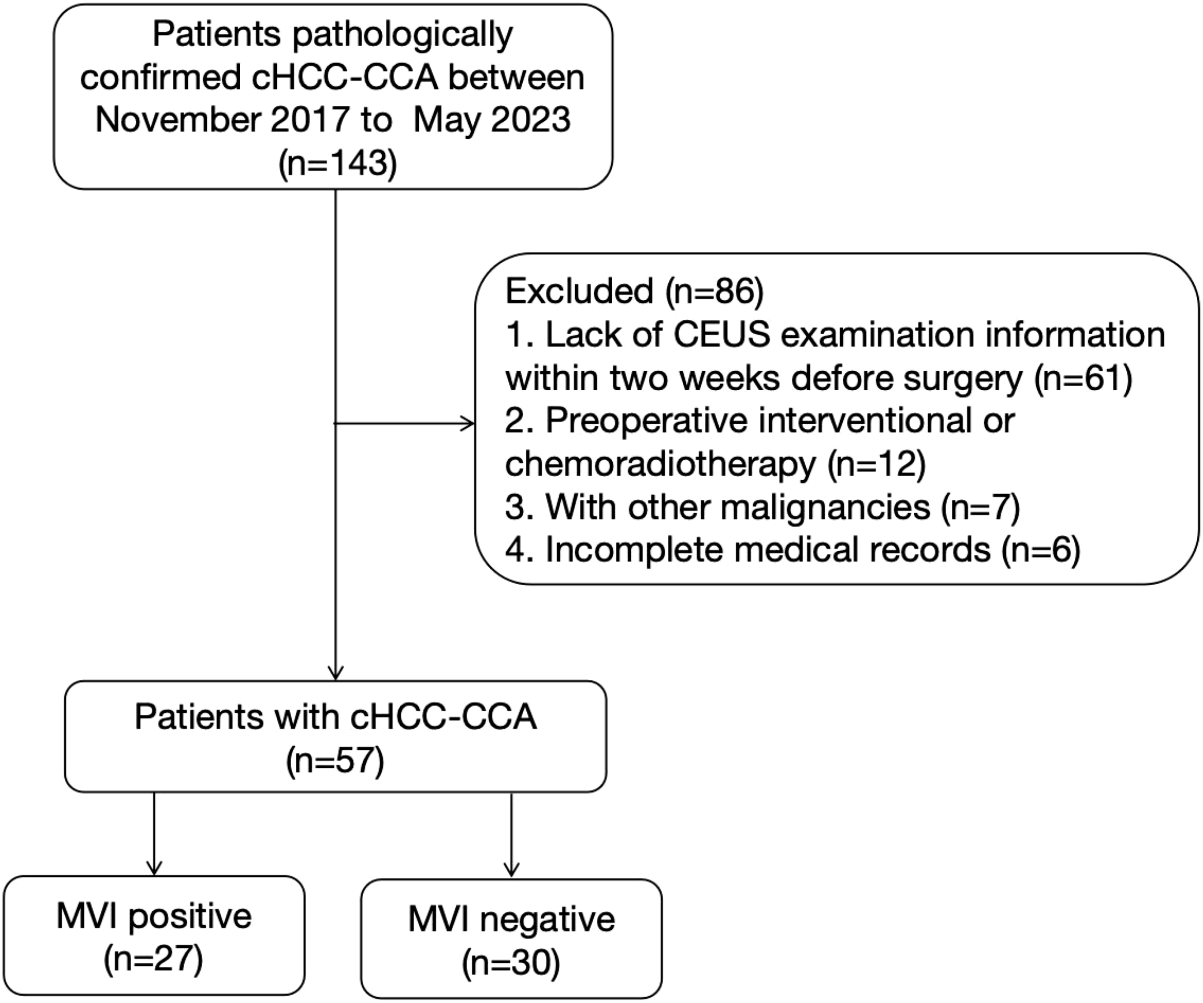
Flowchart of patient selection process. CEUS, contrast-enhanced ultrasound; cHCC-CCA, combined hepatocellular-cholangiocarcinoma; MVI, microvascular invasion.

This retrospective study included 57 cases of cHCC-CCA patients who received medical treatment at Guizhou Provincial People’s Hospital between November 2017 and May 2023, and were confirmed by pathology. The implementation of this study has been approved by the Ethics Committee of our hospital, and informed consent of the subjects has been waived (No: 2024–032). The inclusion criteria were as follows (1): cHCC-CCA confirmed by surgery and pathology based on the 2019 WHO classification (14); (2) undergoing CEUS examination within 2 weeks prior to surgery; (3) complete preoperative clinical data available; (4) presence of MVI information in the postoperative histopathological results. The exclusion criteria were: (1) previous anti-cancer treatments such as local therapy or systemic chemotherapy; (2) presence of concurrent malignancies in other sites; (3) lack of preoperative radiological and clinical data. **Figure 1** illustrates the patient recruitment process.

### 2.2 CEUS techniques

The Aixplorer Sxc6-1 ultrasound system (USA, SuperSonic Imagine) and the Mylab-90 color Doppler ultrasound diagnostic device (Esaote C1-8) with a probe frequency of 1-5 MHz were used. The ultrasound contrast agent SonoVue (Bracco, Italy) was utilized. Before use, it was diluted with 5 ml of 0.9% sodium chloride solution, vigorously shaken, and then injected via the superficial vein of the elbow using a bolus injection method with a volume of 1.5-2.2 ml (determined based on the patient’s weight), followed by a flush with 5 ml of 0.9% sodium chloride solution. The procedure adhered to the guidelines of the Chinese 2017 Ultrasound Contrast Imaging Manual (15). The liver was scanned in the conventional 2D mode to record the number, location, size, borders, internal echoes, and color Doppler blood flow signals of the tumors. Prior to contrast imaging, communication with the patient was conducted to select the optimal position for visualizing the tumor lesions. The contrast agent was injected in the imaging mode, and the timer was started simultaneously. The lesions were continuously monitored for a duration of 5 minutes. If multiple lesions were present, the one with the largest diameter was considered the primary observation target. The contrast images were analyzed and diagnosed by two or more physicians with the rank of associate chief physician or higher.

### 2.3 US and CEUS image analysis

Two professional hepatologists with at least 5 years of experience in liver CEUS analysis retrospectively reviewed ultrasound images without knowledge of the patients’ clinical history and pathological results. Any discrepancies were resolved through consultation with a senior radiologist with more than 10 years of experience. The liver nodules were evaluated based on their enhancement features compared to the surrounding normal liver parenchyma. Clearance refers to tumors showing high enhancement in the arterial phase and low enhancement in the portal or delayed phase. Clearance can be classified into three categories: rapid clearance, where tumor enhancement in the arterial phase is significantly lower than that of the surrounding tissue in the portal or delayed phase; slow clearance, where tumor enhancement in the arterial phase is slightly lower than that of the surrounding tissue in the portal or delayed phase; and no clearance, where tumor enhancement in the arterial phase is consistently not lower than that of the surrounding tissue in the portal or delayed phase. The degree of lesion enhancement is further classified as low enhancement, iso-enhancement, or high enhancement. Enhancement types are subdivided into peripheral irregular rim enhancement, diffuse heterogeneous enhancement, and diffuse homogeneous enhancement. The lesion enhancement types are as follows: (1) peripheral irregular rim-like high enhancement, with irregular rim-like high enhancement around the lesion, uneven low enhancement in the center, and strip-like enhancement extending to the lesion center; (2) diffuse heterogeneous hyperenhancement, with both the periphery and center of the lesion showing heterogeneous hyperenhancement; (3) diffuse homogeneous high enhancement, with both the periphery and center of the lesion showing homogeneous high enhancement. Finally, all liver lesions were classified according to the CEUS LI-RADS (2017 version) (16).

### 2.4 Clinical data and histopathology evaluation

The preoperative clinical data were collected from medical records, including age, gender, history of hepatitis B virus (HBV) or hepatitis C virus (HCV) infection, liver background (presence or absence of liver cirrhosis), tumor markers: alpha-fetoprotein (AFP), carcinoembryonic antigen (CEA), and carbohydrate antigen 19-9 (CA19-9). Pathological results included hematoxylin-eosin staining and immunohistochemical staining, evaluated by two pathologists with 10 years of work experience, who were blinded to the clinical and radiological information. MVI was defined as the invasion of tumor cells into small blood vessels surrounding the tumor, which can only be detected under a microscope. Patients included in the study were divided into MVI-negative and MVI-positive groups based on pathological findings.

### 2.5 Statistical analysis

SPSS 25.0 software (Chicago, IL, USA) was used. Continuous variables with a normal distribution were presented as mean ± standard deviation, and independent samples t-test was used for between-group comparisons. Categorical variables were presented as counts or percentages, and between-group comparisons were conducted using chi-square or rank-sum tests. Multiple-factor logistic regression analysis was employed to identify factors influencing MVI in cHCC-CCA patients. Receiver operating characteristic (ROC) curve was plotted to analyze the predictive value of the influencing factors on MVI. A significance level of P<0.05 was considered statistically significant.

## 3 Results

### 3.1 Clinicopathologic characteristics

This study included a total of 57 patients, of which 47.4% (27/57) were positive for MVI and 52.6% (30/57) were negative for MVI. The clinical and pathological characteristics of the two groups of patients are compared in **Table 1**. There were significant differences between the MVI-positive and MVI-negative groups in terms of tumor size (6.72 ± 3.12 cm vs. 4.29 ± 2.18 cm, p<0.001) and AFP level >400 ng/mL (p=0.046). No significant differences were observed between the two groups in other clinical and pathological data, including age, gender, hepatic background, cirrhosis status, lymph node metastasis, and liver capsule invasion (p>0.05 for all).

**Table 1:** Clinicopathologic characteristics of patients with cHCC-CCA.

Characteristic	MVI-negative (n=30)	MVI-positive (n=27)	*P* value
Age, mean ± SD (years)	57.80 ± 15.91	55.19 ± 10.85	0.413
≤60	18 (60.0)	19 (70.4)	
>60	12 (40.0)	8 (29.6)	
Gender			0.843
Male	17 (56.7)	16 (59.3)	
Female	13 (43.3)	11 (40.7)	
Largest diameter (cm)	4.29 ± 2.18	6.72 ± 3.12	**<0.001**
<5	23 (76.7)	8 (29.6)	
≥5	7 (23.3)	19 (70.4)	
Hepatic background			0.592
Normal	26 (86.7)	22 (81.5)	
Cirrhosis	4 (13.3)	5 (18.5)	
Chronic hepatitis B/C			0.920
Positive	7 (23.3)	6 (22.2)	
Negative	23 (76.7)	21 (77.8)	
Tumor markers			
AFP>20ug/L	9 (30.0)	10 (37.0)	0.574
AFP>400ug/L	2 (6.7)	7 (25.9)	**0.046**
CA19.9>39 U/ml	3 (10.0)	2 (7.4)	0.730
CEA>5 ng/ml	2 (6.7)	2 (7.4)	0.913
Lymph node metastasis			0.355
No	27 (90.0)	22 (81.5)	
Yes	3 (10.0)	5 (18.5)	
liver capsule Invasion			0.091
No	28 (93.3)	21 (77.8)	
Yes	2 (6.7)	6 (22.2)	

The data are expressed as the number (%) of patients.

AFP, alpha fetoprotein; CA19.9, carbohydrate antigen 19.9; CEA, carcinoembryonic antigen.

cHCC-CCA, combined hepatocellular-cholangiocarcinoma; MVI, microvascular invasion.

p<0.05, significant. Bold values indicate statistical significance.

### 3.2 CEUS imaging features

**Figure 2:**
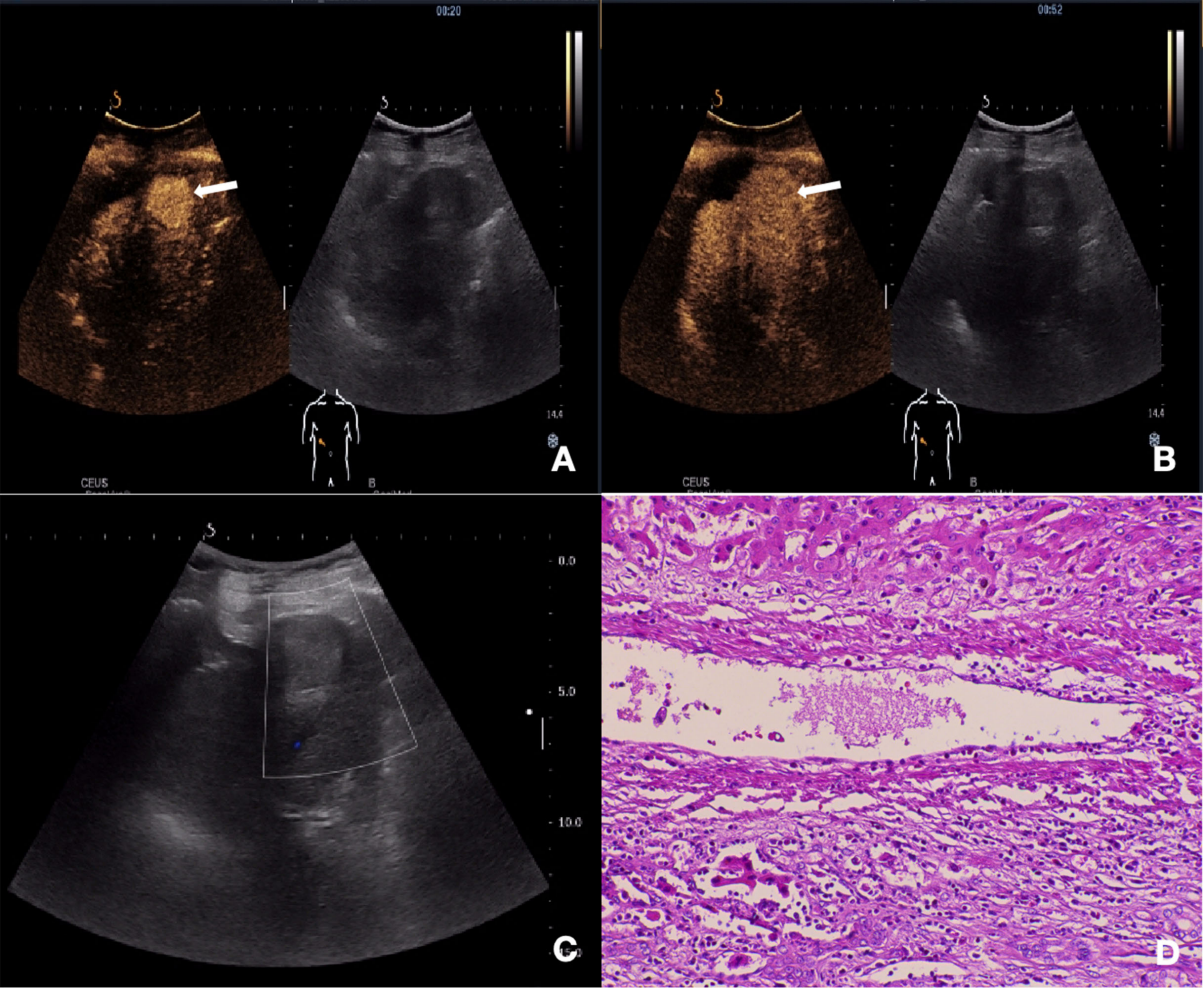
Ultrasonography and contrast-enhanced ultrasound images of cHCC-CCA with negative-MVI. **(A)** CEUS imaging of the mass demonstrates arterial phase homogeneous enhancement (20s post-injection). **(B)** No significant early washout was observed in the portal venous phase (52s post-injection). **(C)** Gray scale ultrasound image shows a lesion in segment III of the liver and Color Doppler showed no obvious blood flow signal. **(D)** Histopathological examination confirmed the diagnosis of cHCC-CCA with negative-MVI (HE staining; ×200).

**Figure 3:**
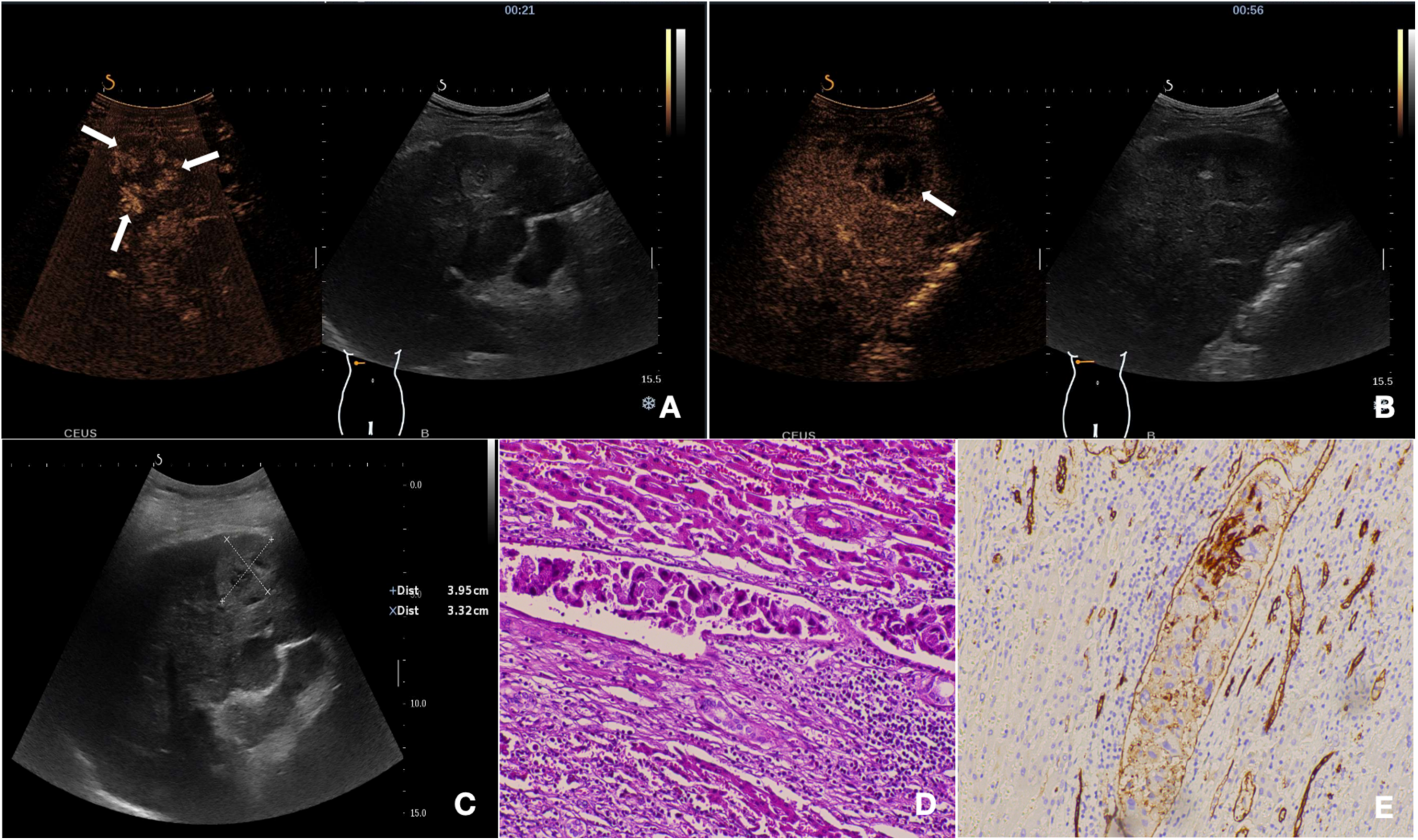
The nodule was pathologically confirmed as cHCC-CCA with positive-MVI. **(A)** The mass showed peripheral irregular rim-like enhancement with central non-enhancement area in the arterial phase on CEUS (21s post-injection). **(B)** The tumor exhibited early washout during the portal venous phase (56s post-injection). **(C)** Baseline ultrasound demonstrates a hypoechoic mass located in segment VI of the liver. **(D)** Histopathological examination confirmed the diagnosis of cHCC-CCA with positive-MVI (HE staining; ×200). **(E)** CD34 labeled vessels were detected by immunohistochemistry (×200).

**Table 2** summarizes the CEUS features of nodules with opposite MVI statuses. Significant differences were observed in the enhancement pattern and washout degree between the two groups (p = 0.007 and 0.013, respectively) (**Figures 2**, **3**). Among the 27 MVI-positive lesions, 16 (59.3%) exhibited a peripheral nodular enhancement pattern, whereas a similar proportion of MVI-negative nodules (56.7%, 17/30) showed homogeneous enhancement. Regarding washout degree, 59.3% of MVI-positive lesions (16/27) demonstrated pronounced washout within 60 seconds, compared to 26.7% (8/30) in MVI-negative nodules. No significant differences were observed between the two groups in terms of other imaging characteristics (all p > 0.05). According to the CEUS LI-RADS (2017 edition) guidelines, 12.3% (7/57) of cHCC-CCA patients were classified as LR-M. However, there was no significant difference in LI-RADS category between the MVI-positive and MVI-negative groups (p = 0.688).

**Table 2:** US and CEUS imaging features of cHCC-CCA.

US and CEUS features	MVI-negative (n=30)	MVI-positive (n=27)	*P* value
Low echo halo			**0.031**
Yes	3 (10.0)	9 (33.3)	
No	27 (90.0)	18 (66.7)	
Tumor contour			**0.006**
Smooth	22 (73.3)	10 (37.0)	
Non-smooth	8 (26.7)	17 (63.0)	
Intrahepatic bile duct dilatation			0.792
Yes	11 (36.7)	9 (33.3)	
No	19 (63.3)	18 (66.7)	
Arterial phase			0.824
Hyperenhancement	11 (36.7)	10 (37.1)	
Iso-enhancement	12 (40.0)	9 (33.3)	
Hypo-enhancement	7 (23.3)	8 (29.6)	
Portal venous phase			0.393
Hyperenhancement	8 (27.7)	8 (29.6)	
Iso-enhancement	18 (60.0)	12 (44.4)	
Hypo- enhancement	4 (13.3)	7 (26.0)	
Equilibrium phase			0.586
Hyperenhancement	3 (10.0)	2 (7.4)	
Iso-enhancement	6 (20.0)	3 (11.1)	
Hypo- enhancement	21 (70.0)	22 (81.5)	
Enhanced patterns			**0.007**
Homogeneous enhancement	17 (56.7)	6 (22.2)	
Heterogeneous enhancement	7 (23.3)	5 (18.5)	
Peripheral irregular Rim-like enhancement	6 (20.0)	16 (59.3)	
Early washout (≤60s)	8 (26.7)	16 (59.3)	**0.013**
LI-RADS category			0.688
LR-3	4 (13.4)	2 (7.4)	
LR-4	9 (30.0)	12 (44.4)	
LR-5	13 (43.3)	10 (37.1)	
LR-M	4 (13.3)	3 (11.1)	

CEUS, contrast-enhanced ultrasound; LI-RADS, Liver Imaging Reporting and Data System. Bold values indicate statistical significance.

### 3.3 Univariable and multivariable analysis

**Figure 4:**
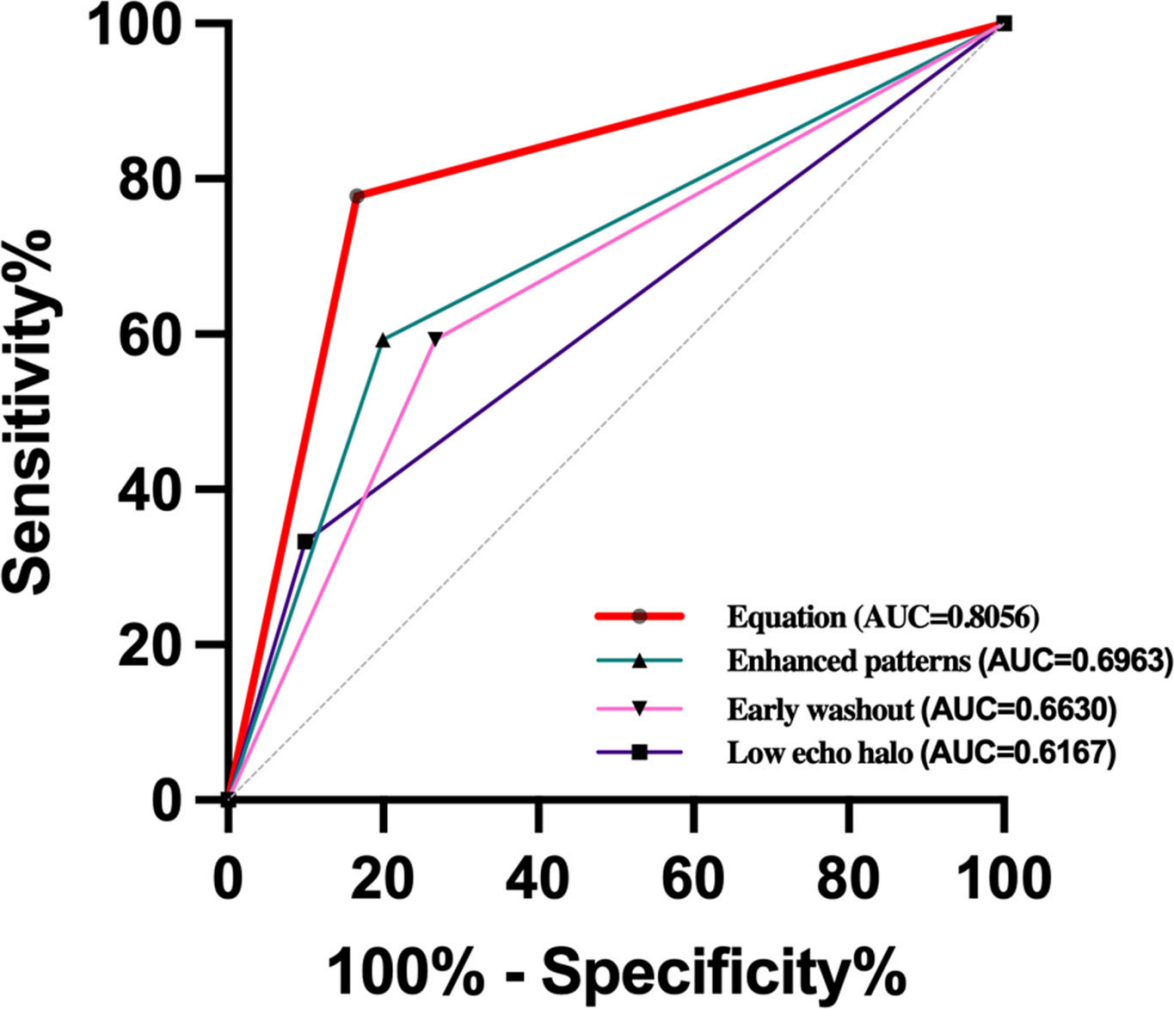
Receiver-operating characteristic (ROC) curves of cHCC-CCA enhanced patterns [area under the ROC curve (AUROC) = 0.6963, early washout (AUROC=0.6630), low echo halo (AUROC=0.6167), and equation (AUROC=0.8056) for the prediction of MVI.

According to the results of univariate analysis in **Tables 1**, **2**, variables with a p-value < 0.05, including tumor size, AFP > 400 ng/mL, low echo halo, non-smooth tumor contour, enhanced patterns on CEUS, and early washout, were included in the multivariable logistic regression analysis. The results showed that low echo halo (OR = 9.602; 95% CI: 1.009, 91.386; P = 0.049), peripheral irregular rim-like enhancement (OR = 8.360; 95% CI: 1.269, 55.056; P = 0.027), and early washout (OR = 10.041; 95% CI: 1.590, 63.412; P = 0.014) (**Table 3**) were independent risk factors for MVI in patients with cHCC-CCA (P < 0.05). Subsequently, a receiver operating characteristic (ROC) curve was constructed, and the results showed that the combined diagnostic value was the highest (AUC = 0.8056) (**Figure 4**).

**Table 3:** Multivariate analyses of risk factors for the MVI of cHCC-CCA.

	OR	95% CI		*P* Value
		Low	Upper	
Largest diameter ≥5 cm	3.660	0.779	17.192	0.100
AFP>400ug/L	3.401	0.397	29.152	0.264
Low echo halo	9.602	1.009	91.386	**0.049**
Tumor contour	2.729	0.520	14.328	0.235
Peripheral irregular Rim-like enhancement	8.360	1.269	55.056	**0.027**
Early washout (≤60s)	10.041	1.590	63.412	**0.014**

Bold values indicate statistical significance.

## 4 Discussion

MVI, as a pathological criterion, refers to the presence of tumor emboli in the blood vessels of the liver tissue adjacent to the tumor. It is mainly observed in the small branches of the portal vein within the tumor-adjacent tissue, and less frequently in the branches of the hepatic vein, hepatic artery, bile duct, and lymphatic vessels, among others (8, 17–19). MVI is an important factor affecting the early postoperative recurrence and disease-free survival rate of cHCC-CCA patients. The presence or absence of MVI determines the treatment approach for cHCC-CCA patients. However, currently, MVI can only be detected under a microscope in surgical specimens or biopsy samples from extensively sampled sites, with a significant lag. Therefore, early identification of MVI helps in formulating the optimal treatment strategy, reducing tumor recurrence, and improving prognosis (20–22). CEUS is an emerging imaging technique for tumor microvascular perfusion information. It offers advantages such as non-radiation, real-time operation, and convenience. However, there is limited research on the preoperative prediction of MVI in cHCC-CCA patients using CEUS (13, 23, 24). This study found a certain correlation between ultrasound and CEUS features and MVI in cHCC-CCA patients, demonstrating important preoperative predictive value.

Our research results indicate that the MVI-positive group and MVI-negative group have statistically significant differences in tumor diameter, AFP level, presence of hypoechoic halo around the tumor, irregularity of nodule margins, tumor enhancement pattern, and early washout time. Multivariate logistic regression analysis incorporating the above indicators reveals that the presence of a hypoechoic halo around the tumor, irregular enhancement of nodule margins, and early washout time are independent risk factors for MVI in cHCC-CCA patients.

Univariate analysis in this study confirms that the tumor diameter is larger in the MVI-positive group compared to the MVI-negative group. Some studies have considered tumor size as a prognostic indicator (25, 26). It is believed that larger tumors have more surrounding liver tissue and, consequently, increased microvessel density, leading to a higher likelihood of MVI. The presence of irregular tumor margins is usually associated with tumor expansion, protrusion beyond the capsule, and invasion into normal liver parenchyma, reflecting the heterogeneity of tumor cell growth and closely correlating with high invasiveness and poor prognosis. Previous studies have shown that irregular tumor margins have a higher sensitivity in predicting MVI in HCC (27). Similarly, in this study, the proportion of MVI-positive group with irregular tumor margins was 63.0% (17/27), significantly higher than the 26.7% (8/30) in the MVI-negative group, providing important reference value for the presence of MVI in cHCC-CCA patients.

Serum AFP is an important serological marker for malignant liver tumors. However, there is still controversy regarding its use in predicting MVI before surgery (28–30). In this study, univariate analysis revealed that patients with MVI-positive cHCC-CCA had higher AFP levels. However, multivariate logistic regression analysis did not support AFP as an independent predictor of MVI in cHCC-CCA. This may be attributed to the inherent heterogeneity of tumors and significant inter-individual differences in AFP levels. In clinical practice, 30%-40% of patients with malignant liver tumors still have negative AFP levels even in the advanced stage of the disease. Therefore, further investigation is needed to determine whether AFP can be used for predicting MVI in cHCC-CCA patients, with a larger sample size.

Previous studies have suggested that the continuous outward growth of tumors can compress the surrounding liver tissue and induce fibrotic reactions, which can be visualized as a hypoechoic halo around the tumor on ultrasound (31–33). This hypoechoic halo has been considered as one of the important factors for predicting MVI. However, whether it can serve as an independent high-risk predictor remains controversial, possibly due to the subjective judgment of the operator and the lack of objective criteria. In this study, it was found that the proportion of tumors with a hypoechoic halo around them was significantly higher in the MVI-positive group compared to the MVI-negative group. Both univariate and multivariate analyses demonstrated that the hypoechoic halo around the tumor was an independent risk factor for MVI. Therefore, when predicting the presence of MVI in patients with cHCC-CCA before surgery, the presence of a hypoechoic halo around the tumor is a relatively important indicator.

The degree of tumor differentiation is correlated with rapid washout (34, 35). Tumors with low differentiation often exhibit rapid clearance, while those with high differentiation show slow regression. This may be due to (1) the remaining normal hepatic sinusoidal tissue in highly differentiated cHCC-CCA, which causes retention of contrast agents due to the presence of orderly trabecular cells and abundant hepatic sinusoids; (2) the growth of nodules is a progressive process. As the malignancy increases and differentiation decreases, abnormal neovascularization and increased blood supply occur. Normal hepatic artery and portal vein blood supply decrease, resulting in a shortened duration of portal enhancement; (3) the more abnormal neovascularization and arteriovenous shunting in the tumor, the shorter the duration of enhancement and the more pronounced portal clearance.

Washout is defined as the visual decrease in enhancement intensity of liver tumors relative to the surrounding liver background in the arterial phase or thereafter, followed by low enhancement. It has two aspects: washout time and washout degree. The degree of washout is classified as marked or mild washout by comparing the enhancement of the nodule with that of the surrounding parenchyma. Zhu et al. (36) explored the washout rate of HCC for predicting MVI on contrast-enhanced ultrasound by reviewing imaging data from 271 HCC patients. Their study indicated a significant correlation between early washout and a high likelihood of MVI. Zhou et al. (37) also found that early washout was an indicator for estimating the occurrence of MVI in HCC in both univariate and multivariate analyses. In contrast, our study found that early washout was an independent risk factor for predicting MVI in cHCC-CCA. The exact mechanism underlying the correlation between washout and MVI remains unclear. This finding may have several explanations. Firstly, tumor microvessel density decreases with the development of MVI, resulting in a reduced dose of contrast agent reaching the tumor site, which leads to attenuation of enhancement and further promotes washout. Secondly, MVI-positive tumors have lower differentiation. Within poorly differentiated tumors, arteriovenous shunting exists, and contrast agents can be completely cleared on contrast-enhanced ultrasound, resulting in a “punched-out” appearance (38, 39). Our study found that early tumor clearance time had significant predictive value for MVI in cHCC-CCA patients in both univariate and multivariate analyses. Therefore, we believe that early tumor clearance time, as a simple and intuitive imaging feature during CEUS, is of great value in predicting the presence of MVI in cHCC-CCA patients. ROC curve analysis showed that hypoechoic halo around the tumor, irregular enhancement of the nodule, and early clearance time had an AUC value of 0.8056 for the combined diagnosis of MVI, providing important diagnostic basis for preoperative prediction of MVI in cHCC-CCA patients.

This study has the following limitations: (1) The study was a single-center retrospective study with possible selection bias. (2)The use of contrast-enhanced quantitative analysis software only allows for the selection of a certain portion of the tumor as the research subject when the tumor volume is large, without conducting quantitative analysis on the entire tumor. (3) Subjective bias exists when dividing the tumor morphology into regular or irregular shapes and determining whether the portal phase is rapidly cleared. (4) Ultrasound examinations are prone to interference from intra-abdominal gas and may lead to the omission of isoechoic lesions.

## 5 Conclusion

There is a certain correlation between the ultrasound and CEUS features and the presence of MVI in patients with cHCC-CCA. Low echo halo around the tumor, peripheral irregular rim-like enhancement of the nodules, and early washout are independent risk factors for MVI in cHCC-CCA patients, which have significant predictive value for the presence of MVI. It provides meaningful reference value for further treatment of patients. However, this study has a relatively small sample size, and multicenter studies are needed to further validate the research findings.

## Data Availability

The original contributions presented in the study are included in the article/supplementary material. Further inquiries can be directed to the corresponding author.
